# Supplementary material for: Atypical Chemokine Receptor CCRL2 Shapes Tumor Spheroid Structure and Immune Signaling in Melanoma
Source: Biomolecules. 2025 Aug 11;15(8):1150. doi: 10.3390/biom15081150 (PMC12384466; doi:10.3390/biom15081150)
Supplement: Supplementary file 1 [file biomolecules-15-01150-s001.zip › Supplementary Figures Al Delbany et al., 2025.pdf]

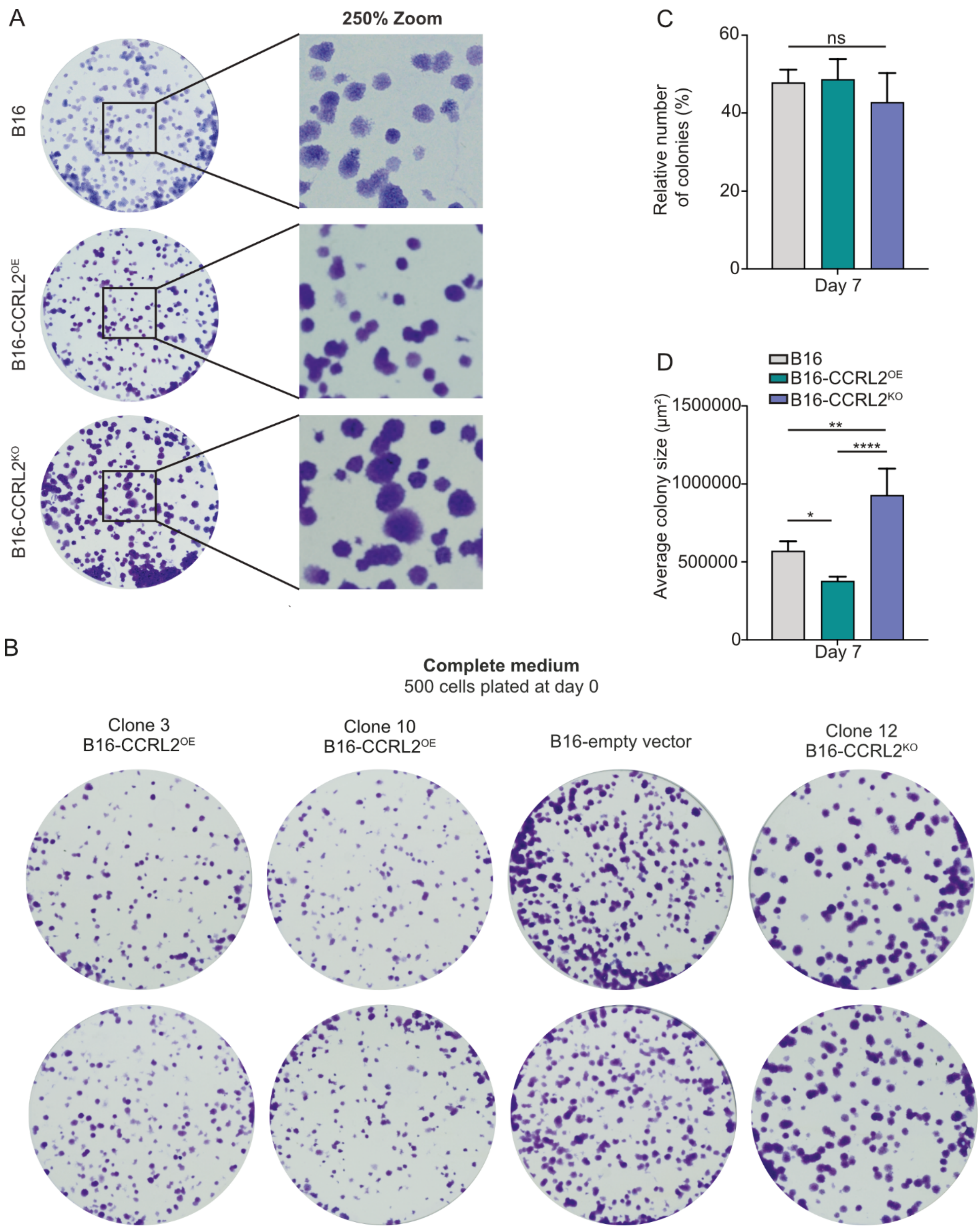

Supplementary Figure S1. **Expanded views and replicate images of clonogenic assays in B16 cells with CCRL2 modulation**. (A) Zoomed-in views (250% magnification) of crystal violet-stained colonies formed by control B16 cells, B16-CCRL2^{OE}, and B16-CCRL2^{KO} cells to highlight differences in colony morphology and density. Insets correspond to enlarged areas from representative wells shown in Figure 2A. (B) Additional representative images from individual B16-CCRL2^{OE} clones (Clones 3 and 10), B16-CCRL2^{KO} clone (Clone 12), and B16 transduced with an empty vector control, plated at 500 cells per well and cultured for 7 days in complete medium. (C) Quantification of colony formation efficiency calculated as: (Number of colonies formed / Number of cells seeded) × 100%. Data are presented as mean ± SD from

two independent experiments. No statistically significant differences were observed among the groups (ns: not significant). (D) Quantification of average colony size (µm²) on day 7. Data represent mean ± SD from two independent experiments. ****p < 0.0001 (one-way ANOVA with Tukey's post hoc test).

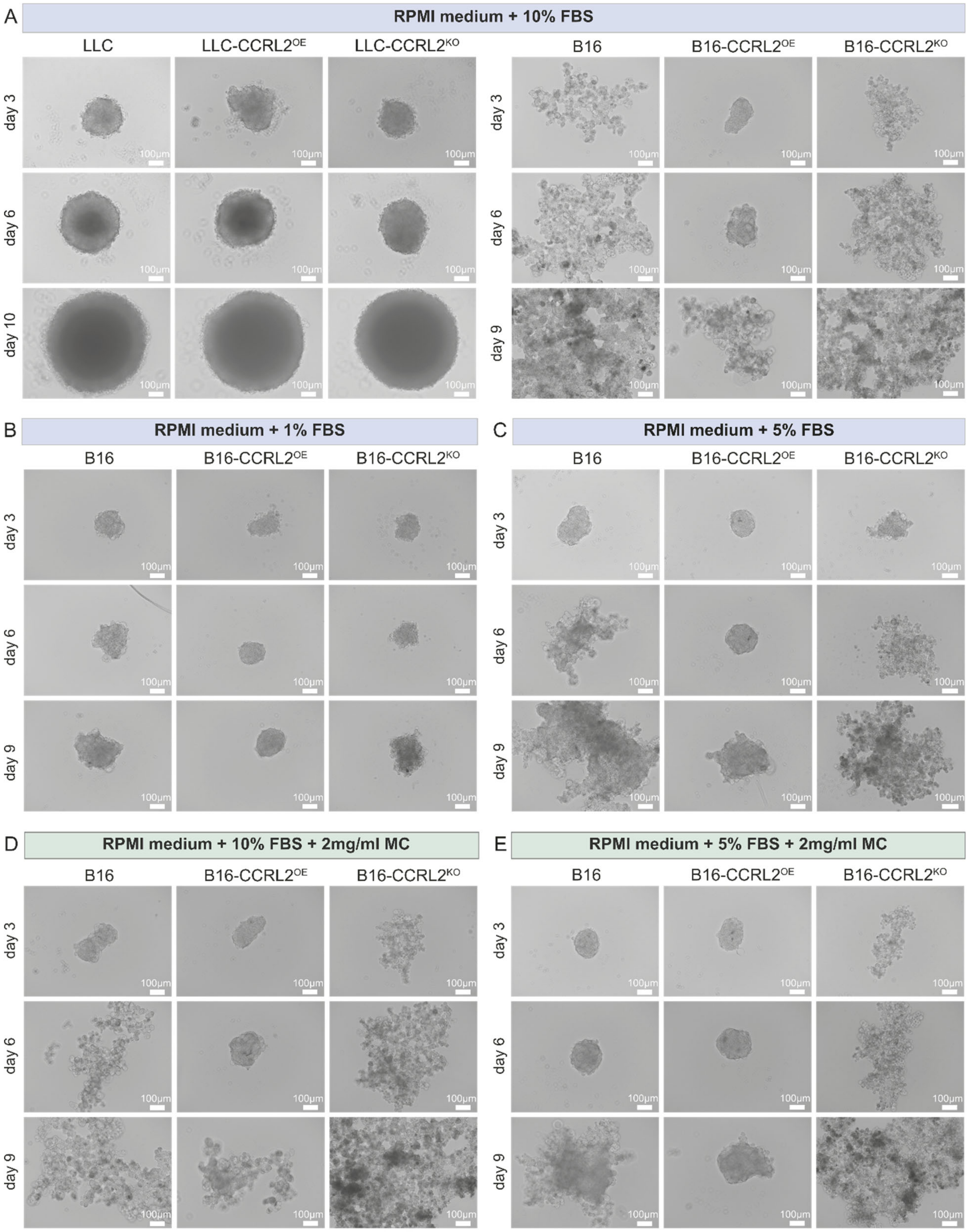

Supplementary Figure S2. Detailed visualization of spheroid formation by B16 melanoma cells under variable culture conditions. (A) Representative images of spheroids formed by LLC and B16 cells at day 6 (control, CCRL2 overexpressing and CCRL2 knockout) cultured in RPMI medium supplemented with 10%

FBS. Images were taken at days 3, 6, and 10 for LLC and days 3, 6, and 9 for B16. (B–C) B16 spheroids grown in RPMI medium containing 1% (B) or 5% (C) FBS. (D–E) B16 spheroids cultured in RPMI medium with either 10% (D) or 5% (E) FBS supplemented with 2 mg/mL methylcellulose (MC), used to enhance aggregation. Scale bars: 100 µm. Images are representative of at least three independent experiments.

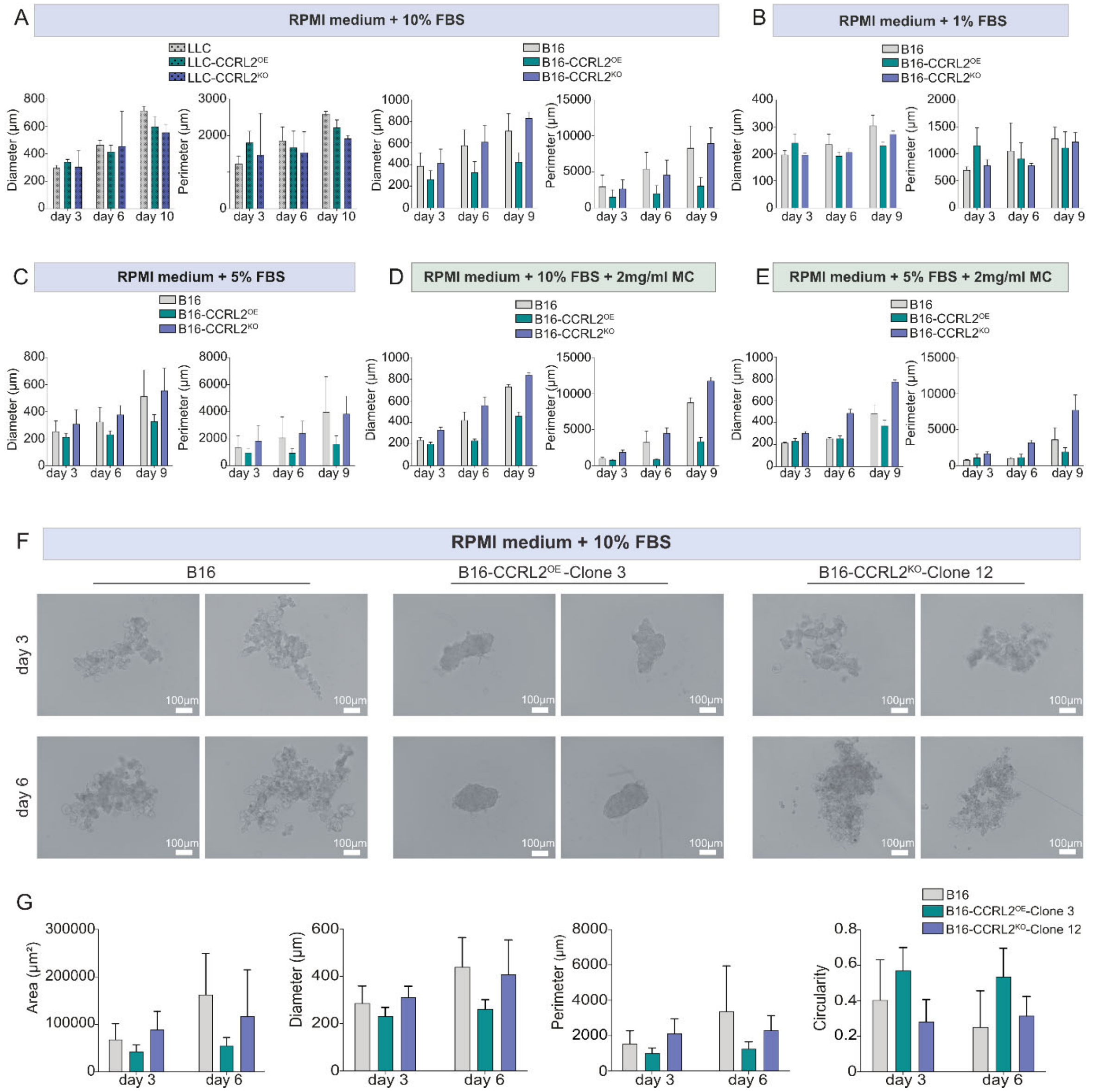

Supplementary Figure S3. Morphometric analysis of spheroid diameter and perimeter in CCRL2-modified LLC and B16 cells under different culture conditions. Spheroid diameter (µm) and perimeter (µm) were quantified using ImageJ in parental (control), CCRL2-overexpressing (CCRL2^{OE}), and CCRL2-knockout (CCRL2^{KO}) variants of LLC and B16 melanoma cell lines. Cells were cultured under varying serum concentrations (panels A–C) or with Methylcellulose supplementation (panels D–E), and measurements were performed at days 3, 6, 9, or 10, depending on the condition. (F) Representative phase-contrast images of spheroids formed by B16, B16-CCRL2^{OE} (Clone 3), and B16-CCRL2^{KO} (Clone 12) cells in RPMI + 10% FBS at days 3 and 6. Scale bars: 100 µm. (G) Quantification of spheroid area (µm²), diameter (µm), perimeter (µm), and circularity for the same B16 clones shown in (F). ImageJ was used for all measurements. All corresponding statistical analyses are provided in Supplementary Table S1.

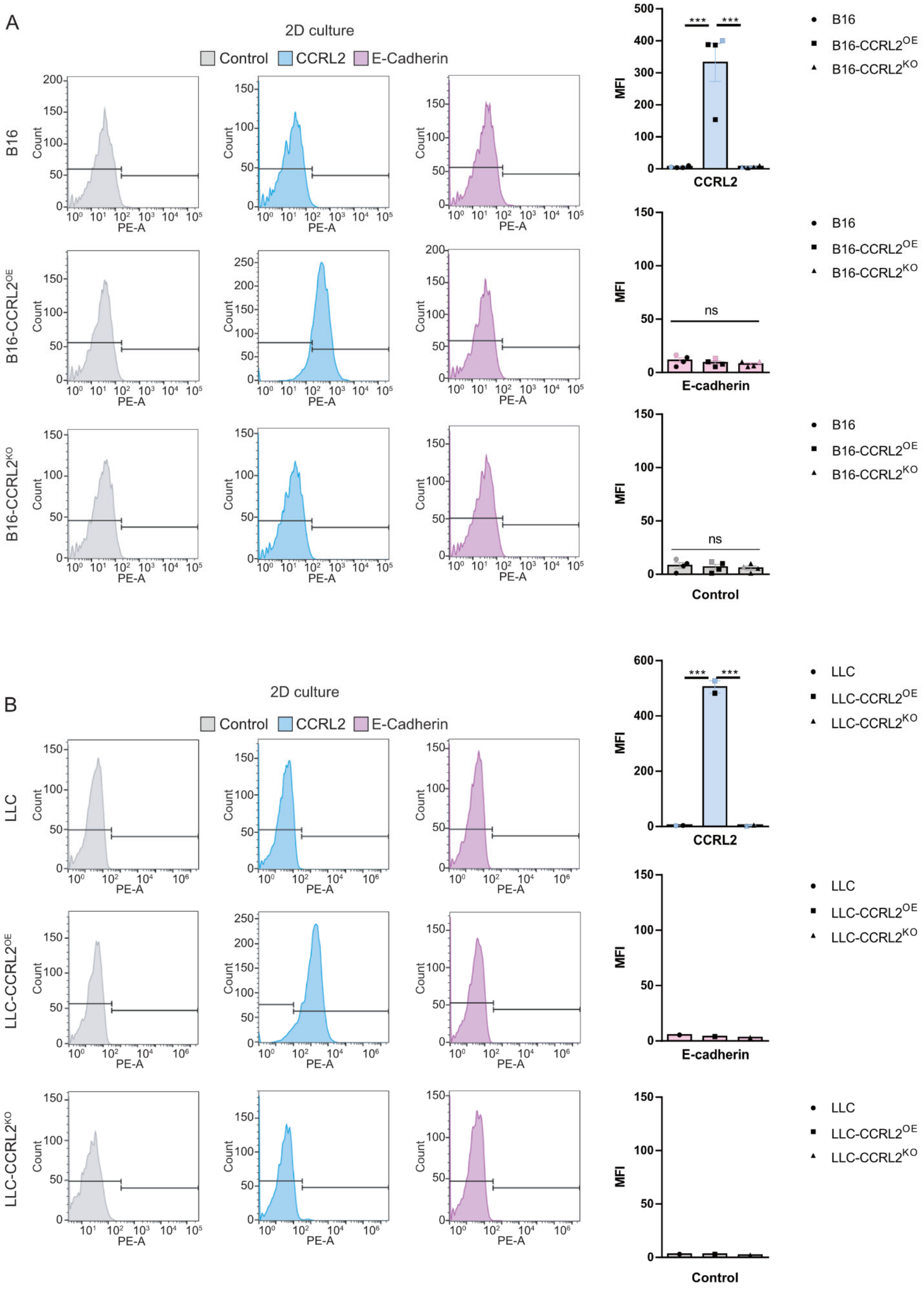

Supplementary Figure S4. CCRL2 and E-cadherin expression in 2D-cultured B16 and LLC cells. (A) Flow cytometry analysis of surface CCRL2 (blue histograms) and E-cadherin (purple histograms) expression in

B16 parental (control), CCRL2-overexpressing (B16-CCRL2^{OE}), and CCRL2-knockout (B16-CCRL2^{KO}) cells cultured in 2D conditions. The corresponding quantification of mean fluorescence intensity (MFI) for CCRL2 and E-cadherin is shown to the right. (B) Equivalent analysis for LLC parental, LLC-CCRL2^{OE}, and LLC-CCRL2^{KO} cells, including histograms (left) and MFI quantification (right). Grey histograms indicate isotype control staining. Statistical significance was determined using one-way ANOVA with Tukey's post hoc test (***p < 0.001; ns: not significant).

A

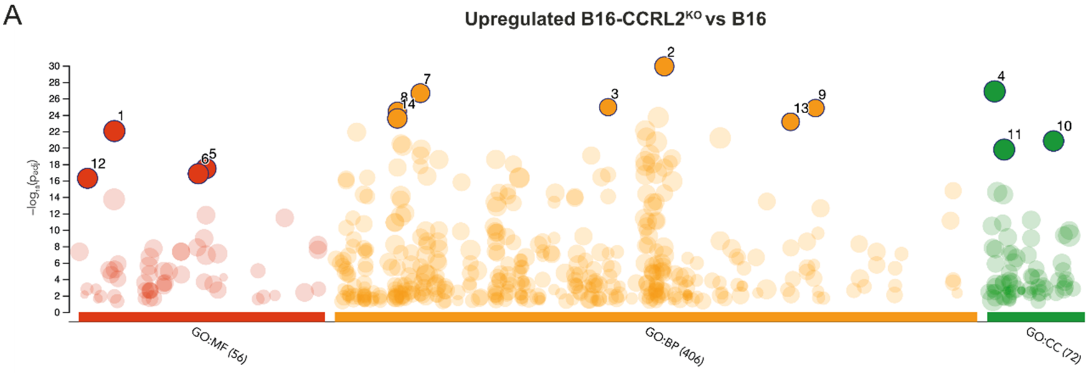

ID	Source	Term ID	Term Name	p_{adj} (query_...
1	GO:MF	GO:0005515	protein binding	9.242×10^{-23}
2	GO:BP	GO:0051239	regulation of multicellular organismal process	1.219×10^{-30}
3	GO:BP	GO:0045087	innate immune response	1.136×10^{-25}
4	GO:CC	GO:0005737	cytoplasm	1.356×10^{-27}
5	GO:MF	GO:0043167	ion binding	3.440×10^{-18}
6	GO:MF	GO:0036094	small molecule binding	1.465×10^{-17}
7	GO:BP	GO:0009605	response to external stimulus	2.243×10^{-27}
8	GO:BP	GO:0006952	defense response	3.563×10^{-25}
9	GO:BP	GO:0140546	defense response to symbiont	1.475×10^{-25}
10	GO:CC	GO:0071944	cell periphery	1.492×10^{-21}
11	GO:CC	GO:0016020	membrane	1.666×10^{-20}
12	GO:MF	GO:0003824	catalytic activity	5.241×10^{-17}
13	GO:BP	GO:0098542	defense response to other organism	6.924×10^{-24}
14	GO:BP	GO:0006950	response to stress	2.680×10^{-24}

B

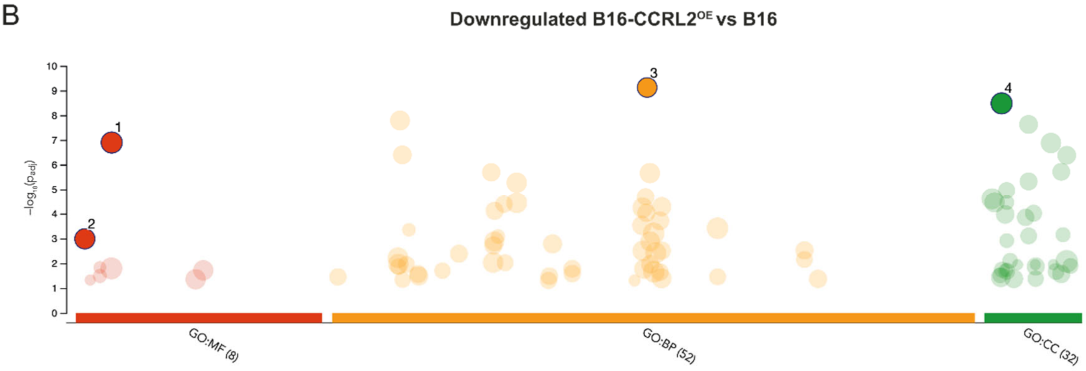

ID	Source	Term ID	Term Name	p_{adj} (query_...
1	GO:MF	GO:0005515	protein binding	1.275×10^{-7}
2	GO:MF	GO:0003824	catalytic activity	1.042×10^{-3}
3	GO:BP	GO:0048731	system development	7.481×10^{-10}
4	GO:CC	GO:0016020	membrane	3.298×10^{-9}

Supplementary Figure S5. GO enrichment analysis of DEGs in B16-CCRL2^{OE} and CCRL2^{KO} cells compared to control B16 cells. GO enrichment analysis was performed using g:Profiler for: (A) upregulated genes in B16-CCRL2^{KO} vs. B16 cells, and (B) downregulated genes in B16-CCRL2^{OE} vs. B16 cells. Each dot represents a significantly enriched GO term, categorized into Molecular Function (GO:MF, red), Biological Process (GO:BP, orange), and Cellular Component (GO:CC, green). The x-axis denotes GO categories, while the y-

axis displays the $-\log_{10}$ of the adjusted p-value. Dot size indicates the number of genes associated with each GO term.
