# Supplementary figures and images for: Atypical Chemokine Receptor CCRL2 Shapes Tumor Spheroid Structure and Immune Signaling in Melanoma
Source: Biomolecules. 2025 Aug 11;15(8):1150. doi: 10.3390/biom15081150 (PMC12384466; doi:10.3390/biom15081150)

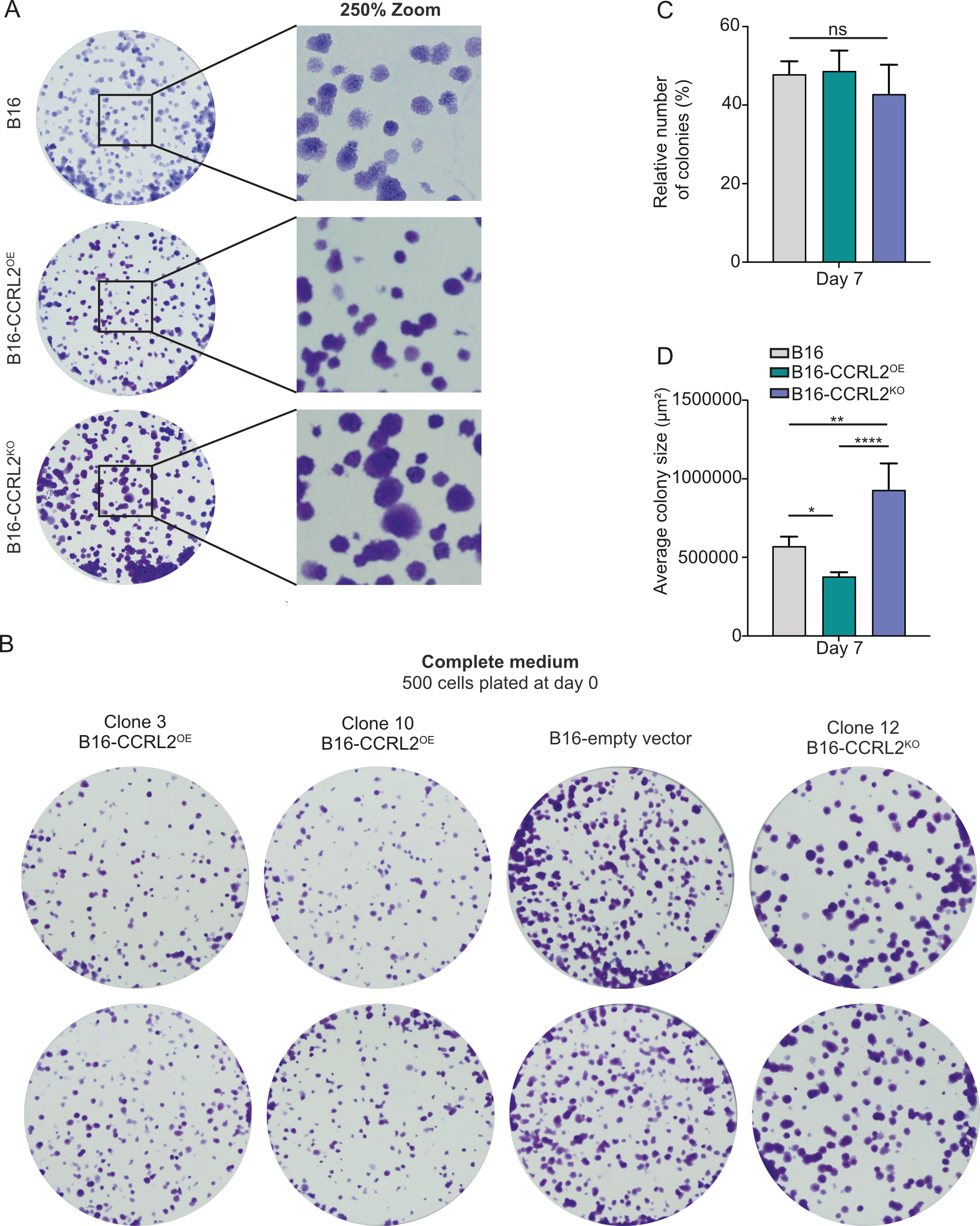

Supplement: Supplementary file 1 [file biomolecules-15-01150-s001.zip › Figure S1.tiff]

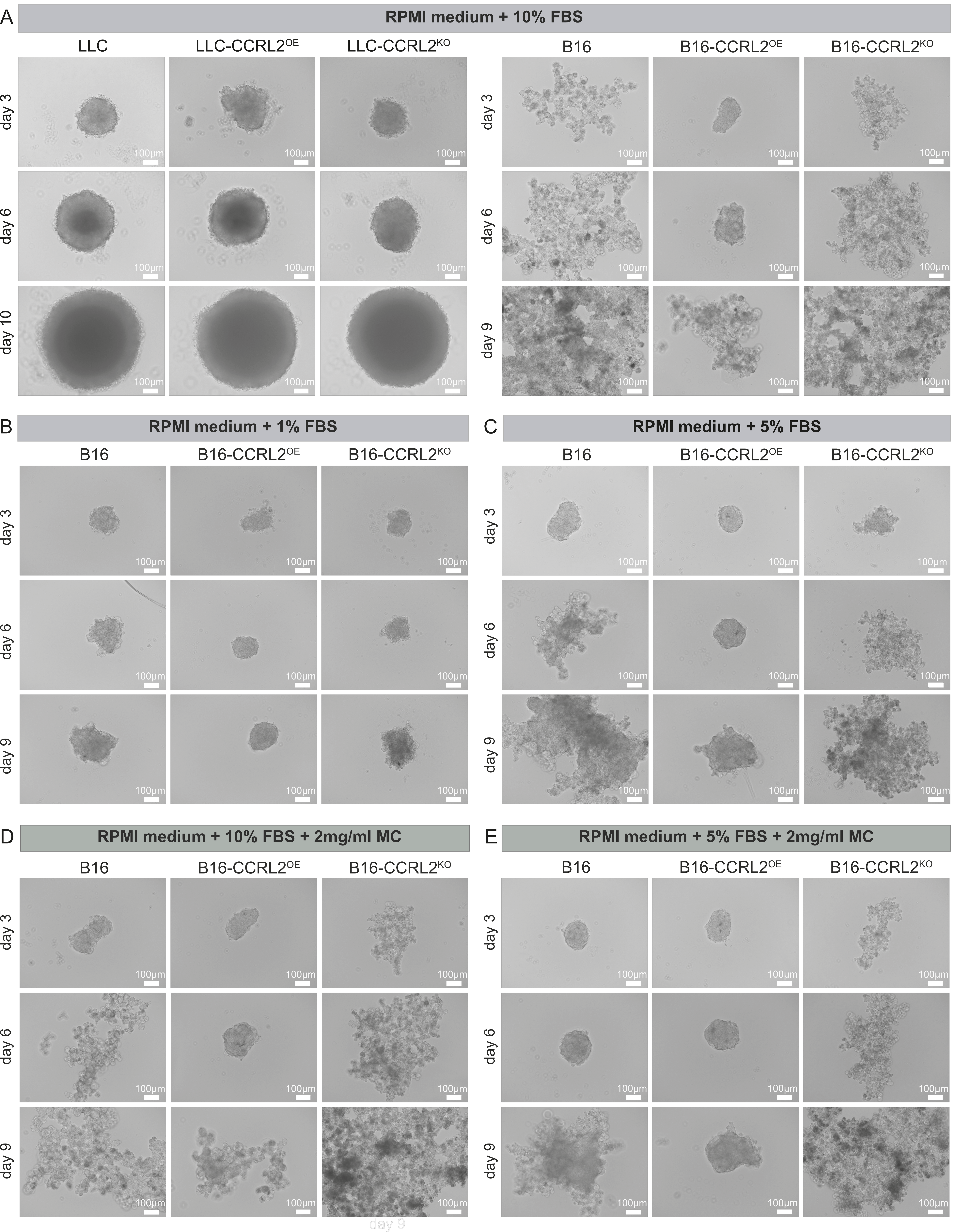

Supplement: Supplementary file 1 [file biomolecules-15-01150-s001.zip › Figure S2.tiff]

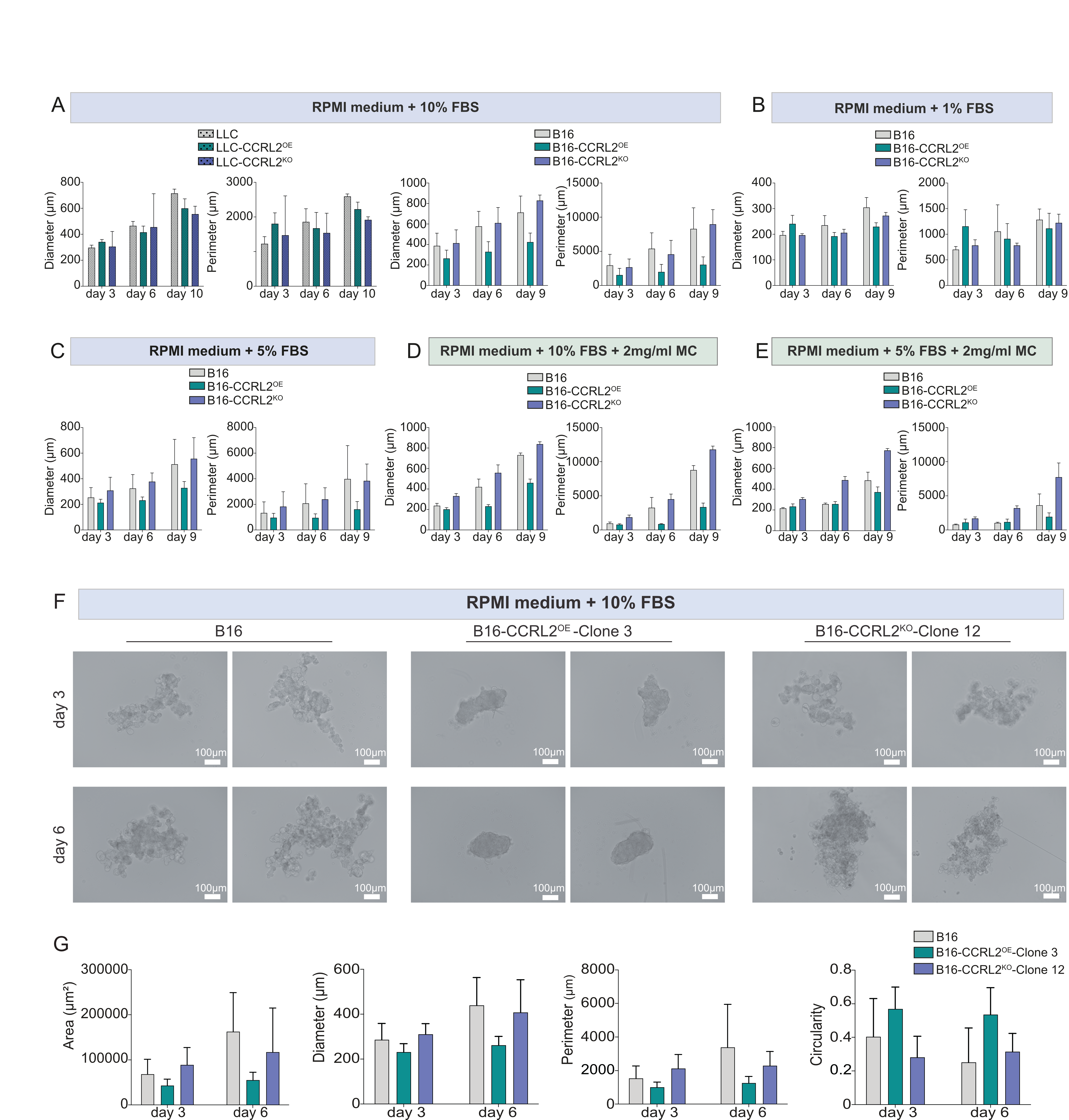

Supplement: Supplementary file 1 [file biomolecules-15-01150-s001.zip › Figure S3.tiff]

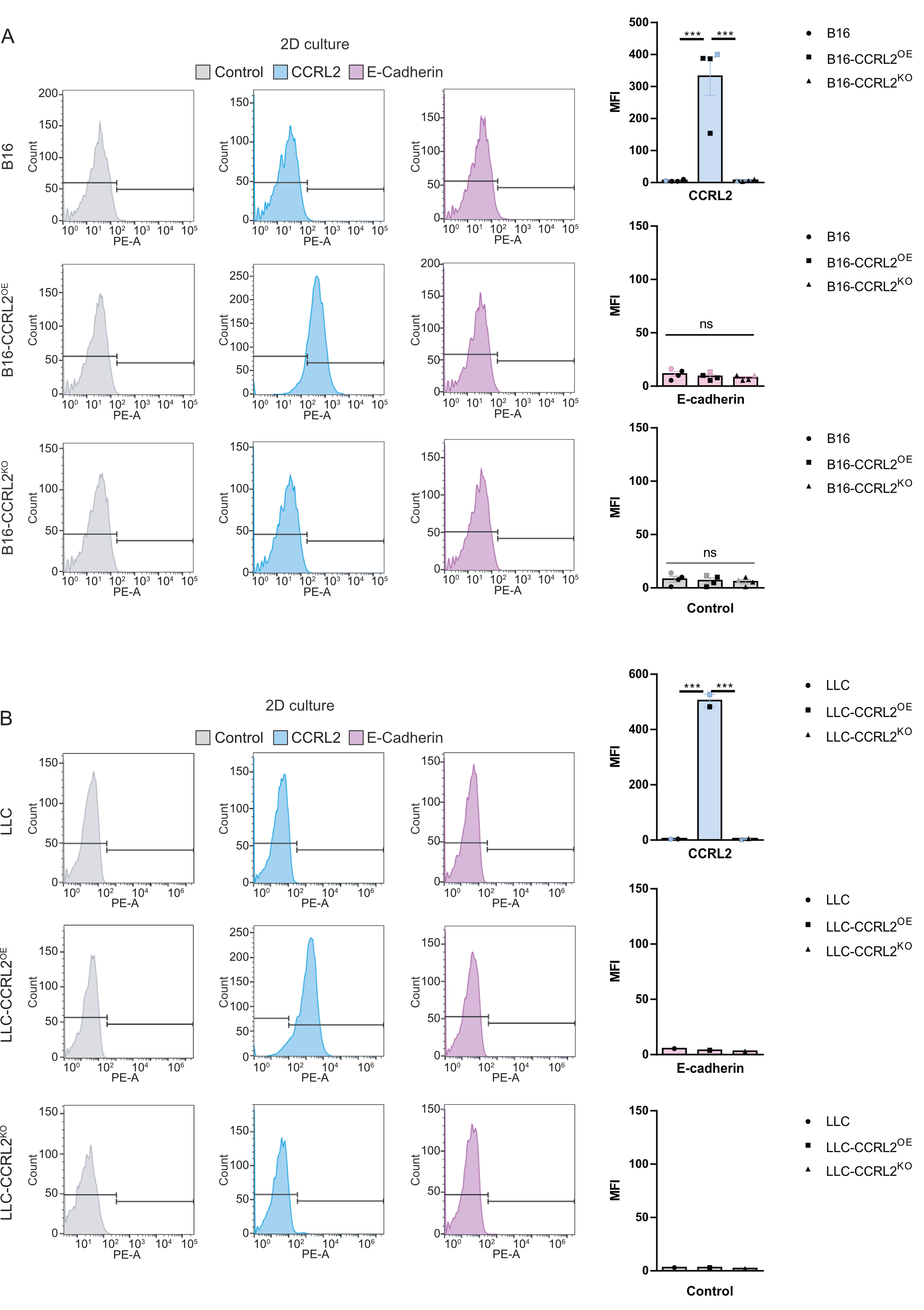

Supplement: Supplementary file 1 [file biomolecules-15-01150-s001.zip › Figure S4.tiff]

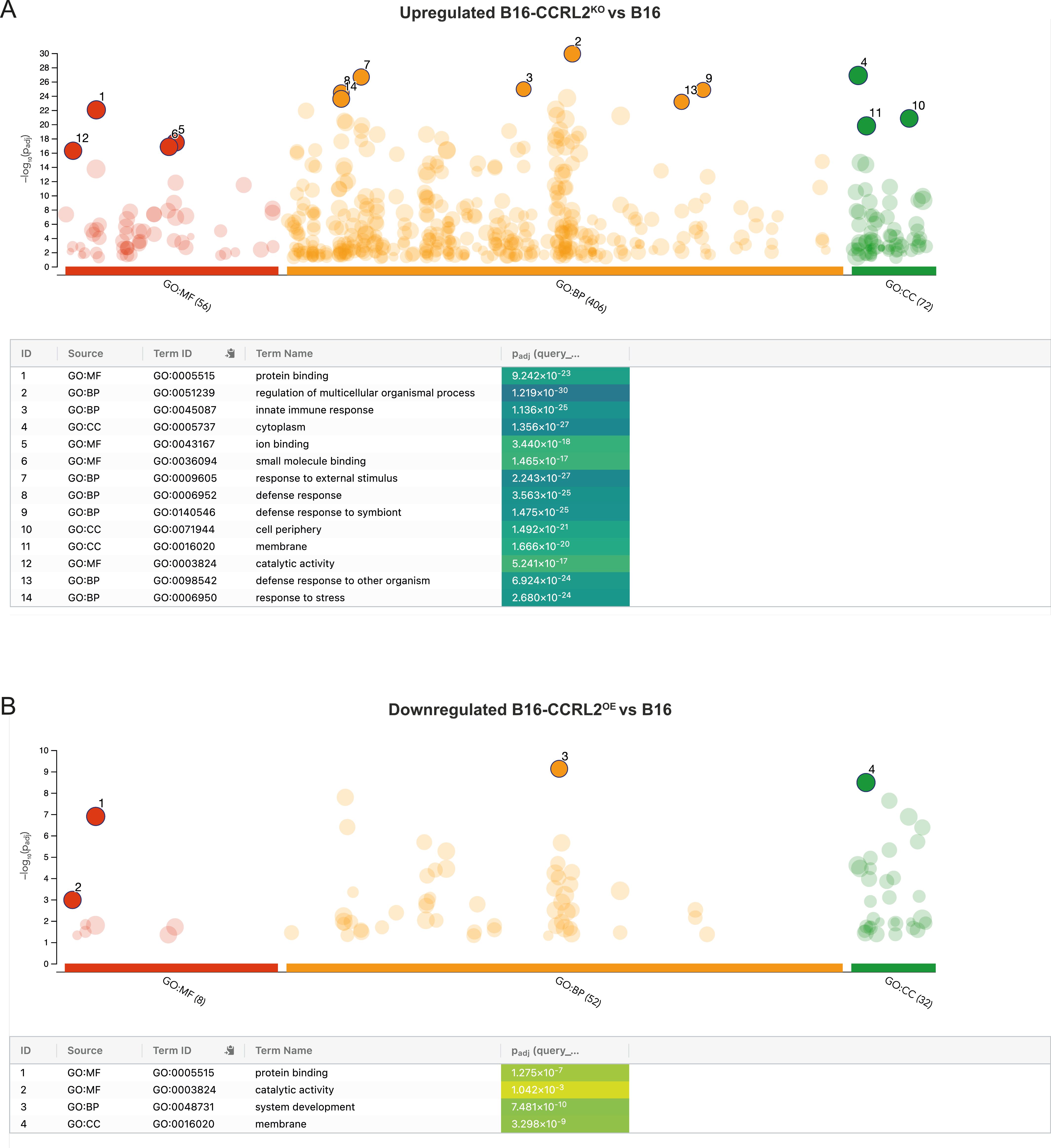

Supplement: Supplementary file 1 [file biomolecules-15-01150-s001.zip › Figure S5.tiff]
